# TRPV3 Channel in Keratinocytes in Scars with Post-Burn Pruritus

**DOI:** 10.3390/ijms18112425

**Published:** 2017-11-15

**Authors:** Chun Wook Park, Hyun Ji Kim, Yong Won Choi, Bo Young Chung, So-Youn Woo, Dong-Keun Song, Hye One Kim

**Affiliations:** 1 Department of Dermatology, Kangnam Sacred Heart Hospital, Hallym University, 1 Singil-ro, Seoul 07441, Korea; dermap@daum.net (C.W.P.); rlatofhal90@hanmail.net (H.J.K.); henric@naver.com (Y.W.C.); victoryby@naver.com (B.Y.C.); 2 Department of Microbiology, School of Medicine, Ewha Womans University, 911-1 Mok-Dong, Yang Cheon-Gu, Seoul 158-710, Korea; soyounwoo@ewha.ac.kr; 3 Department of Pharmacology, College of Medicine, Institute of Natural Medicine, Hallym University, Chunchon 200-702, Korea; dksong@hallym.ac.kr

**Keywords:** protease-activated receptor 2, transient receptor potential vanniloid 3, thymic stromal lymphopoietin, post-burn pruritus

## Abstract

Post-burn pruritus is a common and distressing sequela of burn scars. Empirical antipruritic treatments usually fail to have a satisfactory outcome because of their limited selectivity and possible side effects. Therefore, novel drug targets need to be identified. Here, we aimed to investigate the possible role of protease-activated receptor 2 (PAR2) and transient receptor potential vanniloid 3 (TRPV3), along with the relation of TRPV3 to thymic stromal lymphopoietin (TSLP). Specimens from normal (unscarred) or burn-scarred (with or without pruritus) tissue were obtained from burn patients for this study. In each sample, the keratinocytes were isolated and cultured, and the intracellular Ca^2+^ level at the time of stimulation of each factor was quantified and the interaction was screened. PAR2 function was reduced by antagonism of TRPV3. Inhibiting protein kinase A (PKA) and protein kinase C (PKC) reduced TRPV3 function. TSLP mRNA and protein, and TSLPR protein expressions, increased in scars with post-burn pruritus, compared to scars without it or to normal tissues. In addition, TRPV1 or TRPV3 activation induced increased TSLP expression. Conclusively, TRPV3 may contribute to pruritus in burn scars through TSLP, and can be considered a potential therapeutic target for post-burn pruritus.

## 1. Introduction

Transient receptor potential (TRP) channels are an ion channel group located in the plasma membrane of numerous types of cells. It was shown that certain thermosensitive TRP channels, especially TRP vanilloid 1 (TRPV1), TRPV3, TRPV4 and TRP ankyrin 1 (TRPA1), have important roles in the pathogenesis of pruritus as well as pain [1]. In a few of the early studies, the absence of TRPV3 in the TRP channels of skin cells was reported, but this finding was later reversed [2,3]. Besides skin and neuronal cells, TRPV3 is also expressed in the nasal and oral cavities [4]. In our previous study, we evaluated the clinical and histopathological characteristics of patients with post-burn pruritus and discovered increased expression of TRPV3, TRPV4 and TRPA1 [5]. Among them, TRPV3, in particular, is predominantly expressed in the epidermis of the tissue of pruritic burn scars. Moreover, Ca^2+^ influx via TRPV3 is markedly greater in the keratinocytes isolated from pruritic burn scars than in nonpruritic burn scars or normal skin [6]. In our previous study, protease-activated receptor 2 (PAR2) and neurokinin receptor 1 (NK1R) also were more abundant in pruritic burn scars than in nonpruritic burn scars [6].

Despite these previous studies, there is still much more to be studied about the pathway. For example, how TRPV3, which is highly expressed in keratinocytes, is involved in the itching of burn patients. We hypothesized that the PAR2 pathway might have the effect on TRPV3 of inducing itching in burn scars. This is suggested because of previous discoveries showing that PAR2 activation sensitizes thermoTRPs like TRPV1, TRPV4 and TRPA1 [7,8,9]. Moreover, the effect of PAR2 on TRPV3 channel has not been established.

Thymic stromal lympopoietin (TSLP) is a cytokine involved in allergic diseases and fibrotic conditions such as asthma, allergic rhinitis, atopic dermatitis, systemic sclerosis and scleroderma [10,11,12,13,14]. Intracellular increase of Ca^2+^ promotes TSLP expression through the calmodulin–calcineurin pathway [15]. PAR2 and TRPV1 activation reportedly increased TSLP in airway epithelium [15,16], but the evidence for TRPV3 inducing TSLP expression in epidermal keratinocyte is lacking. Previous study revealed that TSLP derived from keratinocytes act directly via TSLP receptors on sensory nerve terminals to induce pruritus [17]. We hypothesized that epithelial TSLP might have a role in pruritus of burn scar patients and that the TSLP expression level might be increased in burn-scar tissues.

The aims of this study were (1) to determine whether PAR2 downstream pathways have relations with TRPV3 function; (2) to elucidate the mechanism of it and (3) to compare the expression of TSLP in pruritic burn scars with that in nonpruritic burn scars and normal tissue.

## 2. Results

### 2.1. Characteristics of Patients

The 27 burn patients included 19 males and 8 females, ages between 8 and 60 years (36.9 ± 14.2). The average time after burn injury for all patients was 103.2 ± 125.0 months (range, 6–487 months). The patients had total body surface area (TBSA) from 2% to 70% (22.7 ± 22.6). There was no statistical difference on age, proportion of males, TBSA, and time after burn injury (Table 1).

### 2.2. PAR2 Activation Induces Intracellular Ca^2+^ Influx in Cultured Human Keratinocytes and Amplifies TRPV3 Activation Induced Intra + Cellular Ca^2+^ Influx in Burn Scars

To compare intracellular Ca^2+^ influx after TRPV3 activation between keratinocytes cultured from normal tissue and burn-scarred tissue with or without pruritus, TRPV3 agonist (500 μM Carvacrol and 200 μM 2-APB mixture) was used to treat each group.

**Figure 1:**
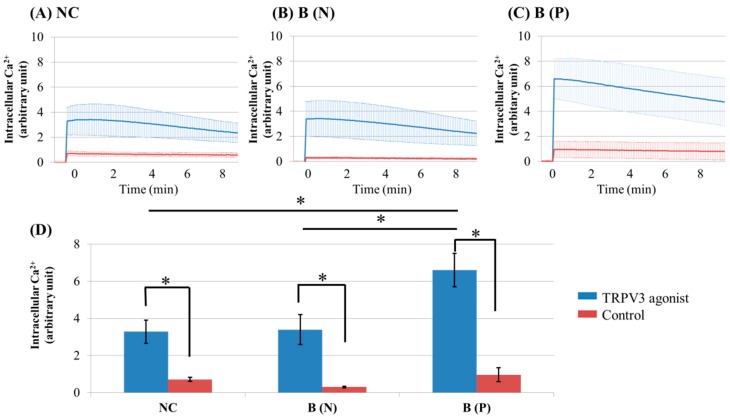
Ca^2+^ influx in cultured keratinocytes of: (**A**) Normal control; (**B**) Nonpruritic burn scar and (**C**) pruritic burn scar; (**D**) Intracellular Ca^2+^ levels at the time of TRPV3 agonist treatment. Each group was treated with TRPV3 agonist (500 μM Carvacrol and 200 μM 2-APB mixture) and Ca^2+^ buffer with 0.5 M ethylenediaminetetraacetic acid (EDTA) was used for control. The Ca^2+^ influx in cultured keratinocytes decreased slowly with time. NC: keratinocytes from normal control; B (N): keratinocytes from a nonpruritic burn scar; B (P): keratinocytes from a pruritic burn scar; Error bars in (**A**–**C**): standard deviation of the mean value obtained from three experiments; Error bars in (**D**): standard error, each performed in triplicate.* *p* < 0.001.

The Ca^2+^ influx in the cultured keratinocytes decreased slowly with time (Figure 1A–C). The keratinocytes from pruritic burn scars showed higher peak level of intracellular Ca^2+^ influx than normal and nonpruritic burn scars, at the time of TRPV3 agonist treatment (Figure 1D).

To investigate the effects of PAR2 on TRPV3 activation, keratinocytes cultured from normal tissue and burn-scarred tissue with or without pruritus, were treated with PAR2 agonist (100 uM SLIGRL-NH2). After that, they were treated with TRPV3 or TRPV1 agonist. The intracellular Ca^2+^ level was recorded continuously with a multimode detector.

**Figure 2:**
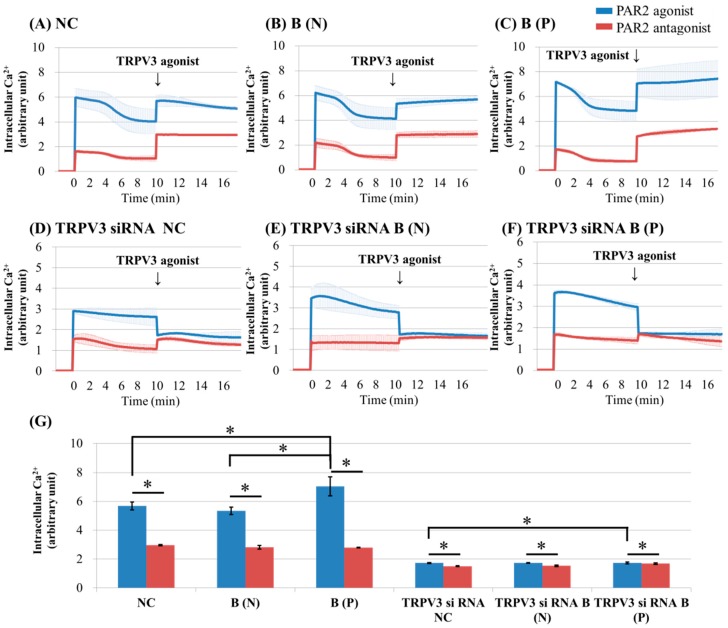
Ca^2+^ influx in cultured keratinocytes of: (**A**) Normal control; (**B**) nonpruritic burn scar; (**C**) pruritic burn scar; (**D**) TRPV3 siRNA transfected normal control; (**E**) TRPV3 siRNA transfected nonpruritic burn scar and (**F**) TRPV3 siRNA transfected pruritic burn scar; (**G**) Intracellular Ca^2+^ levels at the time of TRPV3 agonist treatment. Each group was pretreated with protease-activated receptor 2 (PAR2) agonist (100 μM SLIGRL-NH2) or PAR2 antagonist (100 uM LRGILS-NH2); then TRPV3 agonist (500 μM Carvacrol and 200 μM 2-APB mixture) was added. PAR2 agonist induced intracellular Ca^2+^ influx in cultured human keratinocytes of normal (unscarred), and in nonpruritic and pruritic burn-scar tissues. The pruritic burn scars showed the highest level of intracellular Ca^2+^ influx. Unlike normal tissue, keratinocytes from scar tissue showed a pattern of increasing intracellular Ca^2+^. In keratinocytes transfected with TRPV3 siRNA, PAR2 agonist increased the level of intracellular Ca^2+^, but TRPV3 agonist decreased it. NC: keratinocytes from normal control; B (N): keratinocytes from a nonpruritic burn scar; B (P): keratinocytes from a pruritic burn scar; Error bars in (**A**–**F**): standard deviation of the mean value obtained from three experiments; Error bars in (**G**): standard error, each performed in triplicate.* *p* < 0.001.

**Figure 3:**
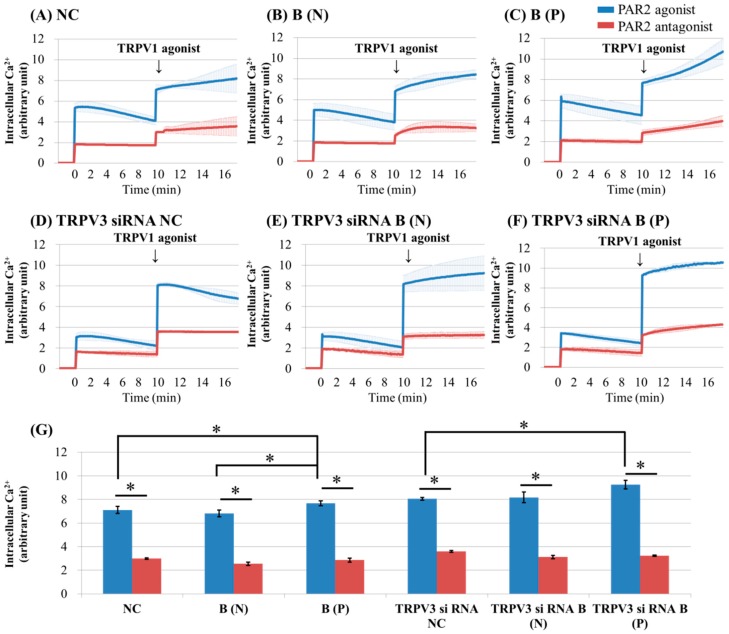
Ca^2+^ influx in cultured keratinocytes of: (**A**) Normal control; (**B**) nonpruritic burn scar; (**C**) pruritic burn scar; (**D**) TRPV3 siRNA transfected normal control; (**E**) TRPV3 siRNA transfected nonpruritic burn scar and (**F**) TRPV3 siRNA transfected pruritic burn scar; (**G**) intracellular Ca^2+^ levels at the time of TRPV3 agonist treatment. Each group was pretreated with PAR2 agonist (100 uM SLIGRL-NH2) or PAR2 antagonist (100 uM LRGILS-NH2); then TRPV1 agonist (1 uM Capsaicin) was added. PAR2 agonist induced intracellular Ca^2+^ influx in cultured human keratinocytes of normal (unscarred), and in nonpruritic and pruritic burn-scar tissues. After treatment with TRPV3 agonist, intracellular Ca^2+^ slowly increased in all keratinocyte groups except for TRPV3 siRNA transfected normal control keratinocyte. NC: keratinocytes from normal control; B (N): keratinocytes from a nonpruritic burn scar; B (P): keratinocytes from a pruritic burn scar; Error bars in (**A**–**F**): standard deviation of the mean value obtained from three experiments; Error bars in (**G**): standard error, each performed in triplicate.* *p* < 0.001.

PAR2 agonist induced intracellular Ca^2+^ influx in cultured human keratinocytes of normal tissue, and in nonpruritic and pruritic burn-scar tissues (Figure 2 and Figure 3). The keratinocytes from pruritic burn scars showed higher peak level of intracellular Ca^2+^ influx than normal and nonpruritic burn scars, at the time of TRPV3 agonist treatment (Figure 2G). Unlike normal tissue, keratinocytes from scar tissue showed an increasing pattern of intracellular Ca^2+^ level (Figure 2A–C). In keratinocytes transfected by TRPV3 siRNA, PAR2 agonist increased intracellular Ca^2+^ level; whereas TRPV3 agonist decreased it (Figure 2D–F).

After treatment with TRPV1 agonist, intracellular Ca^2+^ slowly increased in all keratinocyte groups except for TRPV3 siRNA transfected normal control keratinocyte (Figure 3A–C,E,F). The keratinocytes from pruritic burn scars showed higher peak level of intracellular Ca^2+^ influx than normal and nonpruritic burn scars, at the time of TRPV1 agonist treatment (Figure 3G).

### 2.3. PAR2 Amplification of TRPV3 Agonist Effects Via PKA, PKC and PLC-β Related Mechanism

To examine the mechanism of amplified TRPV3 activation in PAR2 agonist pretreated keratinocytes of burn-scar tissue, we evaluated the level of intracellular Ca^2+^ of cultured keratinocytes from normal tissue or burn-scarred tissue (with or without pruritus) after inhibiting PLC-β, protein kinase A (PKA) and protein kinase C (PKC), which are essential components of intracellular Ca^2+^ influx signalling by PAR2.

**Figure 4:**
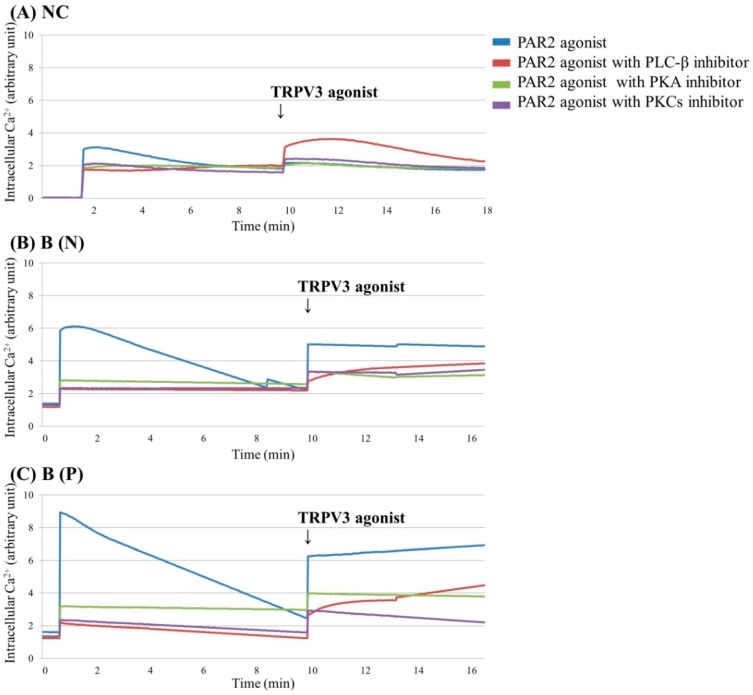
Ca^2+^ influx in cultured keratinocytes of: (**A**) Normal control, (**B**) Nonpruritic burn scar and (**C**) Pruritic burn scar. Each group was pretreated with PAR2 agonist (100 μM SLIGRL-NH2), PAR2 agonist with phospholipase C (PLC)-β inhibitor (10 μM U73122), PAR2 agonist with protein kinase A (PKA) inhibitor (10 μM H-89) or PAR2 agonist with and protein kinase C (PKCs) (α, β, γ, δ, ε and ζ) inhibitor (10 μM GF109203X); then TRPV3 agonist (500 μM Carvacrol and 200 μM 2-APB mixture) was added. Inhibition of PKA and PKC, which are essential components of intracellular Ca^2+^ influx signalling by PAR2, is associated with reduced intracellular Ca^2+^ influx compared to the control, even when treated with PAR2 agonist or with additional TRPV3 agonist treatments. NC: keratinocytes from normal control; B (N): keratinocytes from a nonpruritic burn scar; B (P): keratinocytes from a pruritic burn scar.

The blocking of PKA and PKC is associated with reduced intracellular Ca^2+^ influx compared to the control when treated with PAR2 agonist or with additional TRPV3 agonist treatments (Figure 4).

### 2.4. Inhibition of TRPV3 Channels Attenuated the Action of PAR2

To evaluate the role of the TRPV3 channel on PAR2 function, we compared the intracellular Ca^2+^ level with Fluo-3, with PAR2 agonist in TRPV3 pretreated keratinocytes cultured from normal tissue or burn-scarred tissue (with or without pruritus).

**Figure 5:**
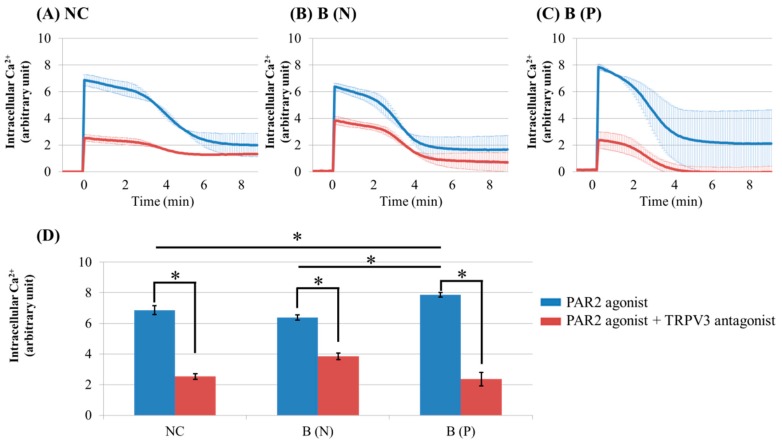
Ca^2+^ influx in cultured keratinocytes of: (**A**) Normal control, (**B**) Nonpruritic burn scar and (**C**) pruritic burn scar. (**D**) Intracellular Ca^2+^ levels at the time of PAR2 agonist treatment. Control group was treated only with PAR2 agonist (100 uM SLIGRL-NH2), while test group was treated with PAR2 agonist and TRPV3 antagonist (125 μM DPTHF). TRPV3 antagonist pretreated group showed lower intracellular Ca^2+^ influx induced by PAR2 agonist than did control group. NC: keratinocytes from normal control; B (N): keratinocytes from nonpruritic burn scar; B (P): keratinocytes from pruritic burn scar; Error bars in (**A**–**C**): standard deviation of the mean value obtained from three experiments, Error bars in (**D**): standard error, each performed in triplicate.* *p* < 0.001.

Intracellular Ca^2+^ influx by PAR2 agonist in keratinocytes was markedly reduced after pre-treatment with TRPV3 antagonist (Figure 5). The keratinocytes from pruritic burn scars showed higher peak level of intracellular Ca^2+^ influx than normal and nonpruritic burn scars, at the time of PAR2 agonist treatment (Figure 5D).

### 2.5. Thymic Stromal Lymphopoietin (TSLP) and TSLPR Expression Increased in Burn-Scar Tissues, Especially in Pruritic Burn Scars

In order to compare the expression of TSLP and TSLPR in normal and burn-scar tissues with or without pruritus, we measured the mRNA level of TSLP in the tissues using qPCR. Then, western blotting was performed to measure the protein level of TSLP and TSLPR in the tissue.

**Figure 6:**
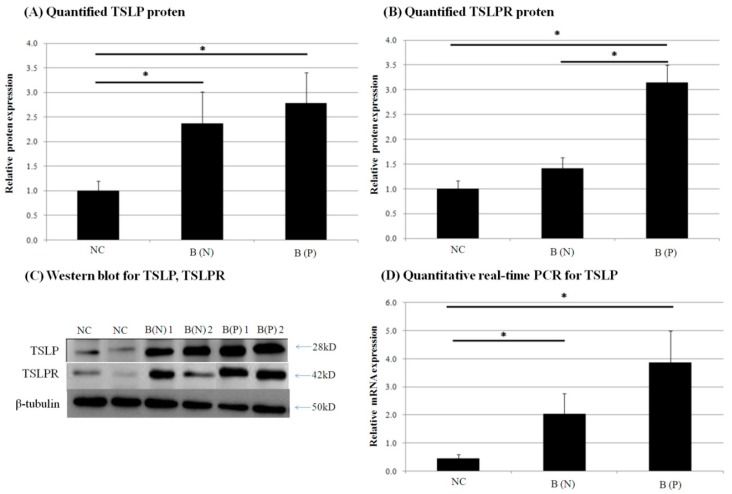
Expression of (**A**) thymic stromal lymphopoietin (TSLP) protein and (**B**) TSLP receptor (TSLPR) protein in normal and burn-scar tissues with or without pruritus was measured by (**C**) western blotting (WB). Expression of (**D**) TSLP mRNA was measured using quantitative real-time PCR in normal and burn-scar tissues with or without pruritus. The greatest TSLP expression of mRNA and protein was observed in pruritic burn-scar tissue using qPCR and western blotting (**A**–**C**). TSLPR proteins were also highest in pruritic burn-scar tissue via western blotting results (**D**). NC: keratinocytes from normal control; B (N): keratinocytes from nonpruritic burn scar; B (P): keratinocytes from pruritic burn scar; TSLP: thymic stromal lymphopoietin; TSLPR: thymic stromal lymphopoietin receptor; Error bars: standard deviation of the mean value obtained from three experiments, each performed in triplicate. * *p* < 0.001.

The highest TSLP expression of mRNA and protein was observed in pruritic burn-scar tissue by qPCR and western blotting. Expression of TSLPR proteins was also greatest in pruritic burn-scar tissue subjected to western blotting (Figure 6).

To visualize the expression pattern of TRPV3, TSLP and TSLPR, immunohistochemistry was performed on normal and burn-scar tissues with or without pruritus, each in twelve sets, using DAPI (4′,6-diamidino-2-phenylindole), TRPV3 antibody, TSLP antibody, TSLPR antibody and PGP 9.5 antibody.

**Figure 7:**
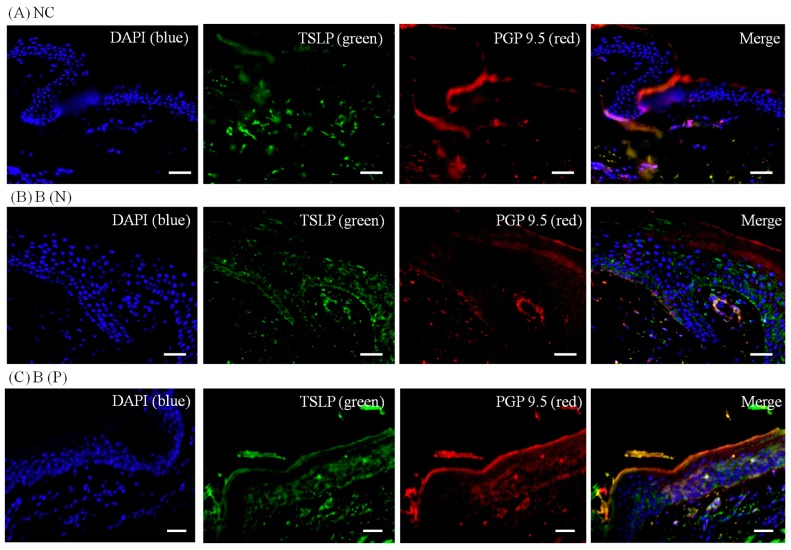
Three-color immunofluorescence confocal images were obtained for 4′,6-diamidino-2-phenylindole (DAPI), **blue**), TSLP (**green**) and protein gene product 9.5 (PGP 9.5, **red**) from tissues of (**A**) normal; (**B**) nonpruritic and (**C**) pruritic burn scars. The staining of TSLP, well observed in the spinous layer of the epidermis, was least in (**A**) normal control and greatest in (**C**) pruritic burn-scar tissue. NC: keratinocytes from normal control; B (N): keratinocytes from a nonpruritic burn scar; B (P): keratinocytes from a pruritic burn scar; TSLP: thymic stromal lymphopoietin. Scale bars = 50 μm.

**Figure 8:**
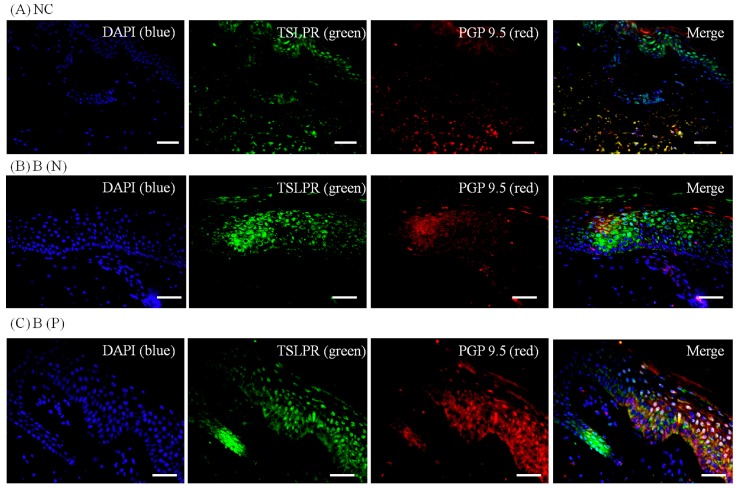
Three-color immunofluorescence confocal images were obtained for DAPI (**blue**), TSLPR (**green**) and PGP 9.5 (**red**) from tissues of (**A**) Normal, (**B**) Nonpruritic and (**C**) Pruritic burn scars. The staining of TSLPR, mainly observed in the lower spinous layer and basal layer of the epidermis, was least in (**A**) Normal control and greater in (**B**,**C**) Burn-scar tissues with or without pruritus. NC: keratinocytes from normal control; B (N): keratinocytes from nonpruritic burn scar; B (P): keratinocytes from pruritic burn scar; TSLPR: thymic stromal lymphopoietin receptor. Scale bars = 50 μm.

**Figure 9:**
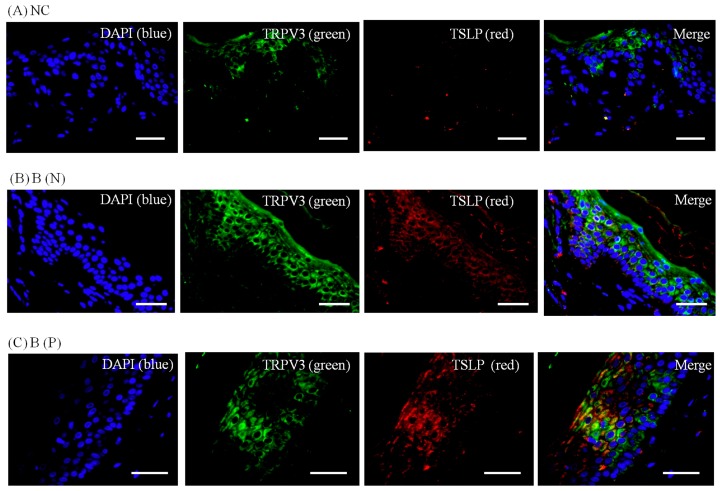
Three-color immunofluorescence confocal images were obtained for DAPI (**blue**), TRPV3 (**green**) and TSLP (**red**) from tissues of (**A**) normal, (**B**) nonpruritic and (**C**) pruritic burn scars. The staining of TRPV3 and TSLP, well observed in spinous layer of the epidermis, was least in (**A**) normal control and greater in (**B**,**C**) burn-scar tissues with or without pruritus. NC: keratinocytes from normal control; B (N): keratinocytes from nonpruritic burn scar; B (P): keratinocytes from pruritic burn scar; TSLP: thymic stromal lymphopoietin. Scale bars = 50 μm.

**Figure 10:**
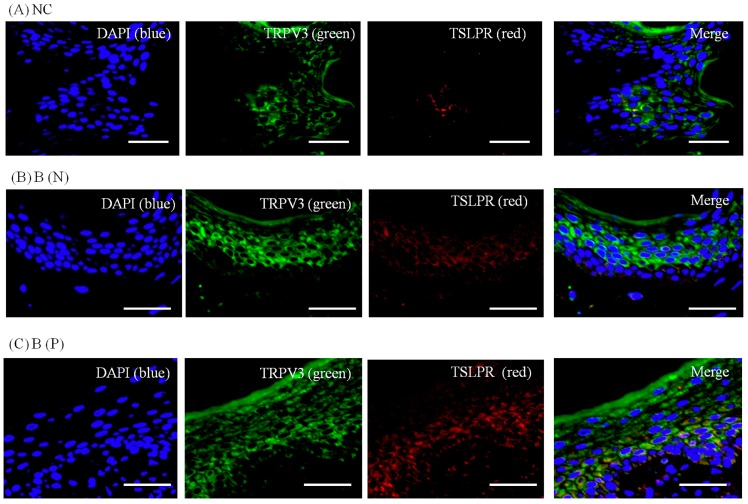
Three-color immunofluorescence confocal images were obtained for DAPI (**blue**), TRPV3 (**green**) and TSLPR (**red**) from skin tissues of (**A**) normal, (**B**) nonpruritic and (**C**) pruritic burn scars. The staining of TRPV3, well observed in spinous layer of the epidermis, and TSLPR, in lower spinous and basal layer, was least in (**A**) normal control and higher in (**B**,**C**) burn-scar tissues with or without pruritus. Staining of TSLPR was greatest in burn-scar tissue with pruritus. NC: keratinocytes from normal control; B (N): keratinocytes from nonpruritic burn scar; B (P): keratinocytes from pruritic burn scar; TSLPR: thymic stromal lymphopoietin receptor. Scale bars = 50 μm.

Immunohistochemistry showed markedly increased TRPV3, TSLP and TSLPR expression in pruritic burn-scar tissue compared to normal (unscarred) tissue. TRPV and TSLP were highly expressed in the spinous layer and TSLPR was mainly observed in the lower spinous layer and basal layer (Figure 7, Figure 8, Figure 9 and Figure 10).

### 2.6. TRPV1 and TRPV3 Activation Induce TSLP Expression in Normal Human Epidermal Keratinocytes

Finally, to confirm that TRPV3 activation induces TSLP production in keratinocytes, normal human epidermal keratinocytes (NHEKs) were transfected with TRPV3 cDNA containing vectors and TRPV3 siRNA. TRPV3 agonist was used to treat MOCK, TRPV3 overexpressing, and TRPV3 blocked NHEKs. TRPV1 agonist was also treated as control. The mRNA level of TSLP was quantified with qPCR, and the protein level was quantified using western blot.

**Figure 11:**
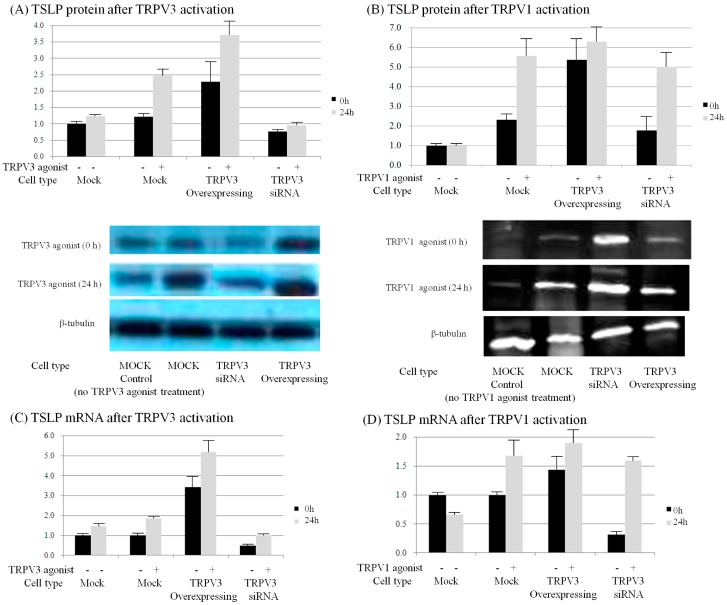
Normal human epidermal keratinocytes (NHEKs) were transfected with TRPV3 overexpressing vector or with siRNA, to analyse the effect of TRPV3 and TRPV1 channels on TSLP expression. Western blotting was conducted to measure the TSLP protein level in response to (**A**) TRPV3 agonist (500 μM Carvacrol + 200 μM mixture 2-APB) and (**B**) TRPV1 agonist (1 μM Capsacin). Quantitative real-time PCR was performed to measure the TSLP mRNA level in response to (**C**) TRPV3 agonist and (**D**) TRPV1 agonist. In normal human epidermal keratinocytes treated with TRPV3 agonist, TSLP mRNA and protein expression increased. The expression of TSLP mRNA was significantly increased by TRPV3 agonist treatment with TRPV3 overexpressed NHEK, but not with *TRPV3* knockout NHEK with siRNA. The expression of TSLP mRNA and protein was also increased by TRPV1 agonist treatment. There was no significant increase in TSLP protein expression in TRPV1 agonist treatment with TRPV3 overexpressed NHEK and no increase in *TRPV3* knockout NHEK. NC: keratinocytes from normal control; B (N): keratinocytes from nonpruritic burn scar; B (P): keratinocytes from pruritic burn scar; NHEK: normal human epidermal keratinocyte; TSLP: thymic stromal lymphopoietin; TSLPR: thymic stromal lymphopoietin receptor; Error bars: standard deviation of the mean value obtained from three experiments, each performed in triplicate.

In normal human epidermal keratinocytes treated with TRPV3 agonist, TSLP mRNA and protein expression increased. The expression of TSLP mRNA was significantly increased by TRPV3 agonist in TRPV3 overexpressed NHEK, but not in the *TRPV3* knockout NHEK with siRNA. The expression of TSLP mRNA and protein was also increased by TRPV1 agonist treatment. There was no significant increase in TSLP protein expression in TRPV1 agonist treatment with TRPV3 overexpressed NHEK, and no increase in the *TRPV3* knockout NHEK (Figure 11).

## 3. Discussion

In this study we demonstrated the relationships of PAR2, TRPV3 and TSLP in keratinocytes from scars with or without pruritus.

PARs are widely expressed G-protein coupled receptors, differentially activated by various physiological factors such as thrombin (PAR1, PAR3 and PAR4), mast cell-derived tryptase (PAR2) and trypsin (PAR2 and PAR4), which mediate various signals [18,19]. PAR2 can be activated by proteases from circulation, inflammatory cells, neurons and keratinocytes; and controls inflammation, pain and neuronal excitability [20,21,22]. Its agonists also cause thermal and mechanical somatic hyperalgesia [23].

In contrast with TRPV1, TRPV4 and TRPA1, which are reportedly sensitized by PAR2 activation in keratinocytes and neurons, there has been no report on the relationship of TRPV3 with PAR2 [7,8,9]. The increase in intracellular Ca^2+^ by PAR2 was greatest in burn scars with pruritus (Figure 2C and Figure 3C). In addition, the effect of PAR2 agonist on intracellular Ca^2+^ (increase) in keratinocytes was not decreased by TRPV3 siRNA treatment (Figure 2D–F); while in the presence of TRPV3 antagonist, the PAR2 agonist was less effective in increasing intracellular Ca^2+^ levels (Figure 5). This suggests that the TRPV3 channel is involved but not necessarily required for PAR2 to increase intracellular Ca^2+^. In the presence of the pretreated PAR2 antagonist in keratinocytes cultured in normal and burn scars, the TRPV3 agonist did not induce marked intracellular Ca^2+^ influx (Figure 2). PLC-β, PKA, and PKC are the subsequent signalling pathways after PAR2 activation. In this study, the action of PAR2 on intracellular Ca^2+^ was decreased by inhibition of PKA and PKC, as expected. Moreover, the action of TRPV3 agonist was also decreased by inhibiting PKA and PKC (Figure 4). In the context of the fact that PKA and PKC modulate the activation of TRPV1, this result implies that PKA and PKC are necessary for TRPV3 to function [24,25,26]. It can be suggested that PKA, PKC and/or other downstream signalling pathways of PAR2 affect TRPV3 inducing Ca^2+^ influx.

On the other hand, the decrease of intracellular Ca^2+^ after treating TRPV3 agonist (carvacrol + 2-APB mixture) on PAR2 agonist pretreated si-RNA transfected keratinocytes (Figure 2D–F) may be caused by the inhibitory effect of 2-APB on inositol phosphate 3 (IP_3_) receptor [27]. The IP_3_ receptor itself is a calcium channel that is linked to intracellular space like endoplasmic reticulum, and when activated, transfers intraluminal calcium to intracellular space to increase intracellular Ca^2+^ level. Inhibition of the IP_3_ receptor, a calcium channel, may have reduced intracellular Ca^2+^. Despite the fact that 2-APB can act on other TRP channels such as TRPV1/2/3/4/6, TRPM7/8, TRPC6, and store-operated calcium channels and show a different pattern in Ca^2+^ regulation in a dose-dependent manner, 2-APB is widely used as TRPV3 agonist since there are few alternatives [28,29,30,31].

Thymic stromal lymphopoietin (TSLP) is known to have an important role in the pathogenesis of atopic dermatitis by mediating an immune response to Th2 [32]. It is released via the Ca^2+^-calmodulin/nuclear factor of activated T cell NFAT pathway in multiple cells including keratinocytes and dendritic cells [33]. Keratinocyte-derived TSLP expression is increased in acute and chronic lesions of atopic dermatitis patients. Genetic variants of TSLP can either induce or protect against the cutaneous inflammation of atopic dermatitis, which may be the result of corresponding increase or decrease in TSLP protein activity [32,34,35]. TSLPR is a heterodimer of the IL-7 receptor alpha chain and TSLP-specific receptor chain, and is expressed in nerve tissue and dorsal root ganglia. We found that TRPV3 and TSLP were increased in burn-scar tissues, especially in burn scars with pruritus (Figure 6 and Figure 7). TRPV3 is responsible for the epidermal layer differentiation and proliferation, which is important to the skin barrier function as well as the pathogenesis of xerotic eczema and the process of wound healing [36,37]. Increased expression of TRPV3 may have a role in the healing of burn injury, and it is possible that increased TRPV3 induced the expression of TSLP. TSLP secreted by keratinocytes may acts directly on TSLP receptors on sensory nerve terminals to induce pruritus [17]. Recently, it has been reported that TSLP expression increased in idiopathic pulmonary fibrosis, atopic dermatitis fibrosis and keloid scar tissue, and it has been reported that it induces transforming growth factor-β, and affects collagen synthesis and fibrocyte infiltration [13]. The findings of our study could be of great significance in explaining the role of TSLP because it might play an important role in the pathogenesis of post-burn pruritus and, although not focused on in this study, burn-scar formation.

The TRPV family of ion channels has six members [38]. Among them, TRPV3 is abundantly expressed in the skin, especially in epidermal and hair follicular keratinocytes, as well as in the cornea, the distal colon, human larynx and inner ear [39,40,41,42,43,44,45,46]. Activation of the epidermal growth factor receptor (EGFR) induces TRPV3 channel activity, which eventually stimulates the release of the growth factor (TGF) [36]. In addition, temperature stimulation activates TRPV3 (and TRPV4) and promotes barrier recovery after mechanical damage [47]. With respect to pain sensation, it is noteworthy that the naturally existing TRPV3 agonists (camphor, eugenol, carvacrol, etc.) function as skin sensitizers, because they induce irritation and pain on topical application [48].

In the present study, expression of TSLP at the protein level and mRNA level increased after TRPV1 and TRPV3 channel activation in normal human epidermal keratinocytes (Figure 11). It has been reported that the activation of the TRPV1 channel in bronchial epithelial cells increases the secretion of TSLP [13]. However, there has been no study of TSLP and TSLPR increase after TRPV1 activation in human epidermal keratinocytes. Furthermore, it is significant that we found that TSLP expression increases after TRPV3 stimulation. Burn-scar tissues had greater TSLP protein and mRNA expression, and greater TSLPR protein expression, than did normal skin tissue (Figure 6). Also, immunofluorescence confocal microscopy images of pruritic burn-scar tissue showed increased staining for TRPV3, TSLP and TSLPR as well (Figure 7, Figure 8, Figure 9 and Figure 10). From the results of our previous study, it appeared that pruritic burn-scar tissue had greater TRPV3 mRNA expression than did normal and nonpruritic burn-scar tissue [5]. Therefore, upregulation of the TRPV3 channel in the scar tissue with pruritus may lead to an increase in TSLP expression, which may be one of the mechanisms of post-burn pruritus.

There have been reports that TRP channels other than TRPV3, such as TRPV1/4, TRPA1, and TRPM6/7 contribute to pruritus. Authors noted at the location of expression of TRP channels, since they are all different. Although TRPV1 and TRPV3 are highly expressed in skin, TRPV1 is also highly expressed in and nerve tissues while TRPV3 is more restricted to the skin, being more selective for itching or pain. Other TRP channels also may have some role in pruritus. However, practically, selective agonists and antagonists have not been found on many TRP channels and further investigation is necessary. Given that TRPV3 is mainly expressed in epithelial tissues, it now appears that agents selectively inhibiting TRPV3 may be beneficial in controlling pruritus and may have fewer side effects.

## 4. Materials and Methods

### 4.1. Patient Selection

Patients with burn scars treated in Hangang Sacred Heart Hospital, Hallym University (Seoul, Korea) were included. Inclusion criteria was burn patients who were 18 years or older who agreed to volunteer. Exclusion criteria were patients with (i) pre-existing chronic systemic or dermatologic diseases which cause pruritus, (ii) concurrent systemic medications affecting pruritus symptoms (such as antihistamines or immunomodulators like systemic steroids and cyclosporine), (iii) concurrent psychotic disease, (iv) pregnancy, and (v) patients who could not provide clinical information about their pruritus. Each patient received a thorough dermatologic examination and underwent a complete examination to exclude other cutaneous or systemic causes of pruritus. Laboratory tests were done to assess blood sugar, liver function and kidney function. The Institutional Review Board of Hangang Sacred Heart Hospital approved the study protocol. Informed consent was obtained from each patient or their parents/legal representative.

### 4.2. Pruritus in Burn Scars

All patients were asked whether they had pruritus on their burn scars. The patients were divided into two groups: those with pruritus and those without.

### 4.3. Tissue Collection

Tissue samples were taken from two different sites from 27 patients undergoing plastic surgery to correct excessive scar tissue: one from a hypertrophic burn scar and the other from normal skin of the inguinal area, which skin is rarely exposed to the sun. In the patients with post-burn pruritus, the specimens were obtained from the most pruritic areas.

### 4.4. Isolation and Cultivation of Keratinocytes

Post-burn hypertrophic scars and normal skin biopsy specimens were obtained. Dispase (Boehringer Mannheim, Mannheim, Germany) (2.4 U/mL for 14 h at 4 °C) was treated to them to mechanically separate the dermis from epidermis. The epidermis part was rinsed with phosphate-buffered saline (PBS) and a single-cell suspension was obtained by treatment with 1 mM EDTA (Gibco, Grand Island, NY, USA) for 30 min in a water bath at 37 °C. The dermis was also washed with PBS and a single-cell suspension was obtained by treatment with 500 U/mL collagenase (Gibco) for 1 h in a water bath at 37 °C. For the cultivation of keratinocytes, isolated cells were rinsed with PBS and were then overlaid (10 × 10^5^/mL) on a culture of adherent, nonproliferating NIH 3T3 mouse fibroblast cells (2 × 10^4^/cm^2^) which is treated with mitomycin C. Cells were cultivated in vitro under standard conditions (5% CO_2_ conditioned atmosphere, 99% humidity and 37 °C) for various time periods before RNA extraction and qRT-PCR were performed. Culture medium of 0.15 mM calcium chloride (3:4 Dulbecco’s modified Eagle’s medium, 1:4 Ham’s F12; Gibco) was supplemented with 10% fetal calf serum (FCS), an antibiotic mixture, 1% l-glutamine, 0.5 μg/mL hydrocortisone, 5 μg/mL insulin, 2.4 μg/mL adenine, 5 μg/mL transferrin, 2 nM triiodothyronine, 1 nM cholera toxin and 10 ng/mL of epidermal growth factor (all from Sigma, St. Louis, MO, USA). Half of the exhausted medium was replaced with fresh medium every 4 days.

### 4.5. Transfecting Normal Human Epidermal Keratinocytes

For TRPV3 siRNA transfection, keratinocytes were cultured for a day in serum-free media. Then, a TRPV3 siRNA reagent system (Santa Cruz Biotechnology, Dallas, CA, USA) was applied. TRPV3 siRNA and the transfection reagent were mixed and incubated for 45 min at room temperature. The TRPV3 siRNA mixture was overlaid onto the washed cell and incubated for 5 h in a CO_2_ incubator. Two-fold serum medium was added and the mixture was cultured for one day.

For TRPV3 overexpression, TRPV3 DNA (TrueORF Gold, Rockville, MD, USA) and Lipofectamine 2000 (Thermo Fisher, Carlsbad, CA, USA) were gently mixed and incubated for 10 min at room temperature. DNA-lipid complex was dripped onto keratinocytes and then cultured for a day in a CO_2_ incubator.

### 4.6. RNA Extraction and Quantitative Real-Time PCR (qRT-PCR)

The total RNA of tissues and cultured cells were extracted and purified using an RNeasy mini kit (#74106, Qiagen, Hilden, Germany) according to the manufacturer’s instructions. A total of 500 ng RNA, and RT premix solution from High Capacity cDNA RT kits (#4374966; Applied Biosystems, Foster City, CA, USA), were added in steps at 25 °C for 10 min, 37 °C for 2 h and 85 °C for 5 min. For the real-time PCR, a reaction mix was prepared containing TaqMan Mastermix (Applied Biosystems), TaqMan probe (Hs00263639_m1 QuantiFast Probe Assay, TSLP; Hs00845692_m1 QuantiFast Probe Assay, TSLPR).

In order to measure TSLP expression in keratinocytes cultured from pruritic, nonpruritic burn scars and normal skin, data was collected using a Light-Cycler480II (Roche, Rotkreuz, Switzerland) under the following conditions: 45 cycles of 95° for 30 s, 60° for 30 s, and 72° for 1 s.

### 4.7. Western Blot

Tissue samples were homogenized in 400 μL RIPA buffer (50 mM Tris-HCl (pH 7.4), 150 mM NaCl, 1 mM PMSF, 1 mM EDTA, 1% Triton X-100, 0.5% sodium deoxycholate and 0.1% sodium dodecyl sulfate (SDS)). The protein was separated on SDS-polyacrylamide gels (20 μg per sample) and transferred to a nitrocellulose membrane using standard procedure (Protran, Schleicher & Schuell, city, Germany). The membranes were put in 5% non-fat milk for 1 h at room temperature and then incubated with primary anti-TSLPR antibody (1:1000; sc-83871, Santa Cruz Biotechnology, Dallas, CA, USA) and anti-TSLP antibody (1:10,000; ab47943, Abcam, Cambridge, MA, USA), overnight at 4 °C. After three washes of 5 minutes each in PBS–Tween 20 (0.1%, *v*/*v*), membranes were incubated with the secondary antibody (1:5000; AP307P, Millipore, Burlington, CA, USA) conjugated with horseradish peroxidase, anti-rabbit IgG for 2 h at 4 °C and then three 5-min washes were performed. The signal was detected with an enhanced chemiluminescence system according to the manufacturer’s manual (Amersham Pharmacia Biotech, Milan, Italy).

### 4.8. Intracellular Ca^2+^ Measurement

Cells (3 × 10^4^/200 mL/well) were cultured in a 96-well plate (NalgeNunc, Naperville, IL, USA) for 3–4 days in supplemented media with 20 μM calcium. Cells were washed using 4-(2-hydroxyethyl)-1-piperazineethanesulfonic acid (HEPES)-buffered saline solution (mM: 121 NaCl, 5.4 KCl, 0.8 MgCl_2_, 25 HEPES, 1.8 CaCl_2_ and 6.0 NaHCO_3_ at pH 7.3) and then loaded with 4 μM Fluo-3/AM (Invitrogen, Carlsbad, CA, USA) with an equivalent volume of 20% Pluronic F127 (Invitrogen) for 45 min in an incubator at 37 °C. Thereafter, the cells were washed twice with HEPES-buffered saline solution for 10 min at room temperature in the dark. Following transfer to EDTA solution with 2 mM calcium at room temperature, the cells were measured using a multimode detector (DTX880, Beckman-Coulter, Brea, CA, USA). Fluorescence was excited by an argon laser at 488 nm and emissions were collected using a 515 filter. The concentrations of materials used for activating and blocking TRP channels in this study were: Carvacrol 500 μM + 2-Aminoethoxydiphenyl borate (2-APB) 200 μM mixture, as TRPV3 agonist; DPTHF 125 μM, as TRPV3 antagonist; Capsacin 1 uM as TRPV1 agonist; SLIGRL-NH2 100 uM, as PAR2 agonist; LRGILS-NH2 100 uM, as PAR2 antagonist; U73122 10 μM, as phospholipase C inhibitor; H-89 10 μM, as PKA inhibitor; GF109203X 10 μM, as PKC-α, β, γ, δ, ε and ζ inhibitor. Chemicals were obtained from Santa Cruz Biotechnology, Sigma, or Abcam.

### 4.9. Confocal Microscopy

Zeiss Axiovert and Bio-Rad MRC1000 confocal microscopes with Zeiss Plan Apo ×40 (NA 1.4) or ×100 (NA 1.3) objectives were used to observe specimens. Image collection was done at a zoom of 1–2, iris of <3 μm and typically 5–10 optical sections were taken at intervals of 0.5 μm. Images were coloured to represent the appropriate fluorophores, and processed using Adobe Photoshop 7.0 (Adobe Systems, Mountain View, CA, USA) to modulate contrast and brightness. Images of stained and control slides were collected and processed identically. The fluorescence intensity of the cells was checked by selecting a straight line across the neuronal soma and using the plot profile function of ImageJ software (version 10.2, NIH image).

### 4.10. Statistical Analysis

Data were expressed as mean ± SD. All statistical analyses were conducted using PASW Statistics 18 (SPSS, Inc., Chicago, IL, USA). Statistical comparisons were made using paired *t*-test or McNemar’s test. Statistical comparisons between two groups were made using the Fisher’s exact test and the Mann–Whitney *U* test.

## Figures and Tables

**Table 1:** The clinical differences between burn patients with and without pruritus.

Characteristic	Nonpruritic Burn Scar Patients (*n* = 15)	Pruritic Burn Scar Patients (*n* = 12)	*p*-Value
Age, years	34.3 ± 23.6	40.1 ± 13.8	0.419
Gender (male), %	80	75	0.6374
Burned area (% of TBSA)	19.3 ± 23.6	26.9 ± 21.4	0.123
Time after burn injury, months	135.6 ± 147.5	62.9 ± 77.9	0.188
VAS for pruritus	0	6.4 ± 0.8	<0.001 *

All data shown are means ± SD or percentage. Significantly different at * *p* < 0.05. VAS, visual analogue scale; TBSA, total body surface area. *p*-values were calculated by Wilcoxon signed rank test for continuous variables and chi-square test for categorical variable.
